# Foxp3^+^ Tregs are recruited to the retina to repair pathological angiogenesis

**DOI:** 10.1038/s41467-017-00751-w

**Published:** 2017-09-29

**Authors:** Devy Deliyanti, Dean M. Talia, Tong Zhu, Mhairi J. Maxwell, Alex Agrotis, Jack R. Jerome, Emily M. Hargreaves, Steven Gerondakis, Margaret L. Hibbs, Fabienne Mackay, Jennifer L. Wilkinson-Berka

**Affiliations:** 1 Department of Diabetes, The Central Clinical School, Monash University, Melbourne, VIC 3004 Australia; 2 Department of Immunology and Pathology, The Central Clinical School, Monash University, Melbourne, VIC 3004 Australia; 3 Biomedicine Discovery Institute and Department of Biochemistry and Molecular Biology, Monash University, Clayton, VIC 3800 Australia; 4 School of Biomedical Sciences, The University of Melbourne, Parkville, VIC 3052 Australia

## Abstract

Neovascular retinopathies are major causes of vision loss; yet treatments to prevent the condition are inadequate. The role of regulatory T cells in neovascular retinopathy is unknown. Here we show that in retinopathy regulatory T cells are transiently increased in lymphoid organs and the retina, but decline when neovascularization is established. The decline is prevented following regulatory T cells expansion with an IL-2/anti-IL-2 mAb complex or the adoptive transfer of regulatory T cells. Further, both approaches reduce vasculopathy (vaso-obliteration, neovascularization, vascular leakage) and alter the activation of Tmem119^+^ retinal microglia. Our in vitro studies complement these findings, showing that retinal microglia co-cultured with regulatory T cells exhibit a reduction in co-stimulatory molecules and pro-inflammatory mediators that is attenuated by CTLA-4 blockade. Collectively, we demonstrate that regulatory T cells are recruited to the retina and, when expanded in number, repair the vasculature. Manipulation of regulatory T cell numbers is a previously unrecognized, and promising avenue for therapies to prevent blinding neovascular retinopathies.

## Abstract (web summary)

The local immune responses in the eye are attenuated to preserve sight. Surprisingly, Deliyanti et al. show that regulatory T cells (Tregs) take an active role in protecting the eye from neovascularization in oxygen-induced retinopathy, and that interventions that augment the retinal Treg numbers reduce neovascular retinopathy in mice.

## Introduction

Increasing evidence indicates that inflammation has a critical role in the pathogenesis of neovascular retinopathies, including retinopathy of prematurity, diabetic retinopathy, and age-related macular degeneration^1–3^. Retinopathy of prematurity is the main ocular disorder of the premature infant and a significant cause of vision loss and blindness that is increasing in prevalence^2, 4^. The hallmark feature of this disorder is progressive damage to the microvasculature in the inner region of the retina. This pathology is initiated by the cessation of developmental angiogenesis (vaso-obliteration) due to the delivery of supplemental oxygen to premature infants to overcome respiratory distress. Following the withdrawal of supplemental oxygen, the retina becomes ischemic and attempts to reinstate developmental angiogenesis by markedly upregulating the production of pro-angiogenic factors such as vascular endothelial growth factor (VEGF). This compensatory new blood vessel growth (neovascularization) is excessive and the fragility of this vasculature results in the exudation of fluid as well as hemorrhages that compromise vision. The factors that predispose neonates to develop retinopathy of prematurity include not only supplemental oxygen (retinal hyperoxia followed by ischemia)^5^, low birth weight and low gestation age^6–8^, but there is growing evidence that exposure to infection and inflammation in the antenatal and prenatal periods increase the risk of developing the disorder^4, 9–11^.

For decades the mainstay treatment for retinopathy of prematurity has been laser photocoagulation to remove areas of neovascularization and vascular leakage as well as surrounding ischemic tissue^2^. However, laser photocoagulation does not prevent the advancement of damage to the microvasculature and is associated with injury to the healthy retina. Of significant interest is the contribution of microglia to retinal vascular pathology^12, 13^. Microglia are close relatives of macrophages of the innate immune system, and the resident immunocompetent cell of the central nervous system^12, 14, 15^. Microglia are a plastic cell population that roam the retina to protect neighboring cells from injury by the release of neuroprotective factors and phagocytosis of cell debris^12, 13^. In response to chronic insults, such as that occurring in retinopathy of prematurity, microglia become activated and release pro-inflammatory mediators that promote damage to the retinal vasculature and stimulate neovascularization^13, 16, 17^. It is not entirely clear if macrophages also contribute to retinopathy of prematurity, as previous methods to distinguish microglia from macrophages have inherent limitations^18^. Moreover, the factors regulating the activation of microglia and macrophages in the retina are not fully understood, and it is likely that other immune cells participate in this disorder.

T regulatory cells (Tregs) expressing the Forkhead box P3 (Foxp3) transcription factor are a key component of the adaptive immune system and play a critical role in immune homeostasis and self-tolerance through their powerful immunosuppressive properties^19^. The potency of Tregs is due to their ability to migrate to tissues and dampen inflammation including the activation of macrophages^20–22^. This occurs by a variety of means such as by cell-to-cell contact and the release of suppressive cytokines^23–25^. These actions of Tregs have resulted in considerable interest in the potential of harnessing Tregs as an immunotherapy for various diseases^26–28^. However, the concept that Tregs migrate to the retina and influence the development of retinopathy of prematurity has not been evaluated. This may be due to the conventional view that in most circumstances the retina is largely an immune privileged site^29^ as well as the previous difficulty in interrogating the Treg compartment in a small and delicate tissue such as the neonatal retina.

We hypothesized that Tregs are recruited to the retina, but that their abundance is reduced during the development of neovascularization in retinopathy of prematurity. Therefore, we sought to determine if expanding the number of Tregs attenuated retinal vasculopathy and if this involved a change in the activation state of microglia in the retina. Our studies were performed in a robust and well characterized model of retinopathy of prematurity known as oxygen-induced retinopathy (OIR)^2, 30^. Here we show that the expansion of Treg numbers results in their penetration into retinal tissue and a reduction in vision-threatening retinopathy, including vaso-obliteration, neovascularization, and vascular leakage. These results indicate that harnessing the immunosuppressive capacity of Tregs is a potential therapy for the treatment of neovascular retinopathies.

## Results

### Treg abundance is altered in OIR and retina

**Fig. 1:**
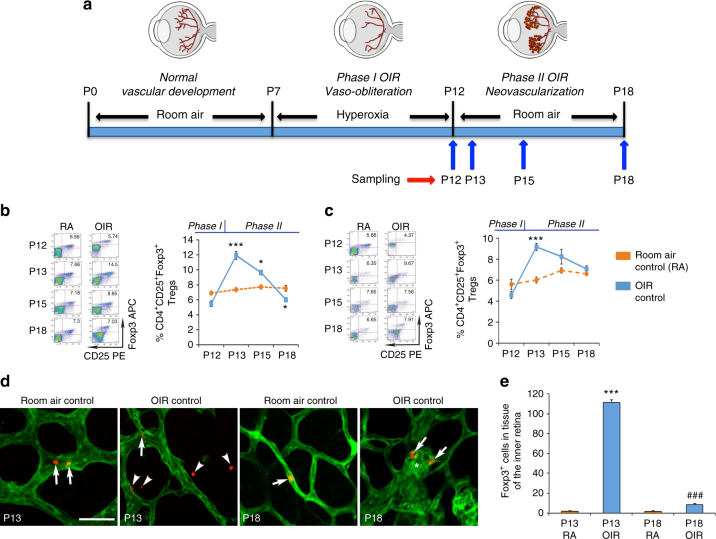
Tregs are transiently increased in OIR and penetrate the retina. **a** Cohorts of C57BL/6J mice were studied during phase I OIR at postnatal day (*P*) 12 and phase II OIR at P13, P15, and P18. Comparisons were made to age-matched room air controls (RA). FACS of pooled lymph nodes **b** and spleen **c** revealed that Foxp3^+^ Tregs were increased in phase II OIR at P13 and declined to the level of RA or below by P18 (enlarged FACS plots in Supplementary Fig. 2). Fluorochromes are phycoerythrin (PE) and allophycocyanin (APC). *n = *13 RA mice at P13 and P18. *n* = 7 OIR mice at P13 and P18. *P < 0.05 and ***P < 0.001 to age-matched RA (one-way ANOVA with Mann–Whitney *U* test). **d** Representative confocal images of flat mounted retina from Foxp3^rfp^ mice at P13 and P18 labeled with isolectin (*green*) to show blood vessels. *Scale bar*, 50 μm. In RA, Foxp3^+^ cells (*red*) were mainly located within blood vessels (*arrows*). In OIR at P13, Foxp3^+^ cells were increased and mainly located in retinal tissue (*arrowheads*). In OIR at P18, the number of Foxp3^+^ cells had declined and were localized to tufts of neovascular blood vessels (*asterisk*) with few cells in retinal tissue. **e** Foxp3^+^ cells in tissue of the inner retina (one-way ANOVA with Mann–Whitney *U* test). ***P < 0.001 to all groups. ^###^ *P < *0.001 to RA. *n = *16 RA mice and OIR mice at P13. *n* = 16 RA mice and 12 OIR mice at P18. Values are expressed as mean ± s.e.m

We first evaluated if in OIR any changes in Treg abundance in lymphoid organs and retina might associate with the disease. OIR occurs in two phases in the developing retina and involves tissue hyperoxia followed by tissue ischemia^2^. In phase I, the exposure of neonatal mice to hyperoxia between postnatal days (P) 7 to P12 mimics the clinical situation when some preterm infants receive supplemental oxygen to assist breathing^2^. In phase I OIR, extensive vaso-obliteration occurs in the central retina and is maximal at P12 due to a suppression of pro-angiogenic factors^2, 30^. The subsequent exposure of mice to room air for ~1 week comprises phase II OIR, which induces retinal ischemia and the excessive production of pro-angiogenic factors that cause marked pathological neovascularization and vascular leakage in the inner retina^2, 30^ (Fig. 1a). We analyzed the frequency of Tregs by flow cytometry (FACS) in pooled lymph nodes and spleens and expressed the data as the percentage of CD4^+^ T cells rather than in absolute numbers due to OIR mice having lower body weights^31^ and organ weights compared to controls (Supplementary Table 1 and Supplementary Fig. 1). In the hyperoxia and retinal vaso-obliterative stage of phase I OIR at P12, Treg frequency in all lymphoid organs analyzed were similar to age-matched room air controls (Fig. 1b, c and Supplementary Fig. 2). However, in the acute ischemia of phase II OIR at P13, the abundance of Tregs in lymphoid organs markedly increased, declining thereafter to the level of room air controls by P18 (Fig. 1b, c and Supplementary Fig. 2).

An important next step was to determine if Tregs penetrated the retina. Due to the increase in Treg numbers in lymphoid organs at the commencement of tissue ischemia at P13, we examined this time point and also P18 when neovascularization and vascular leakage are extensive. As the study of small numbers of Tregs in the neonatal retina is technically challenging by FACS, we ran OIR models using Foxp3^rfp^ mice engineered to express a red fluorescent protein (RFP) specifically in Tregs under the control of the *Foxp3* promoter^32^. We used confocal microscopy on flat mounted retina to quantitate Foxp3^+^ cells, an analysis that focused on the inner retina since this is the main site of vascular damage in OIR^2^. Therefore, this examination excluded the outer retina comprised of photoreceptors, the retinal pigment epithelium, and choroid. In room air controls, few Foxp3^+^ cells were located in retinal tissue with most residing within blood vessels and in similar amounts at P13 (29.7 ± 1.5, all values are mean ± s.e.m.) and P18 (25.8 ± 1.1) (Fig. 1d, e). OIR at P13 resulted in a marked increase in the number of Foxp3^+^ cells in retinal tissue (Fig. 1d, e), and a similar number of these cells were within blood vessels compared to room air controls (24.5 ± 2.8). By P18 OIR, Foxp3^+^ cells had declined in number in retinal tissue compared to P13 OIR (Fig. 1d, e) and were located within blood vessels (22.5 ± 1.0) in similar numbers to room air controls.

These findings suggest that the acute retinal ischemia of OIR is a strong stimulus for the expansion of the Treg compartment and their recruitment into retinal tissue, but that Tregs reach the retina in numbers that are insufficient to repair the vasculature.

### Expanding Treg numbers increased their abundance in retina

**Fig. 2:**
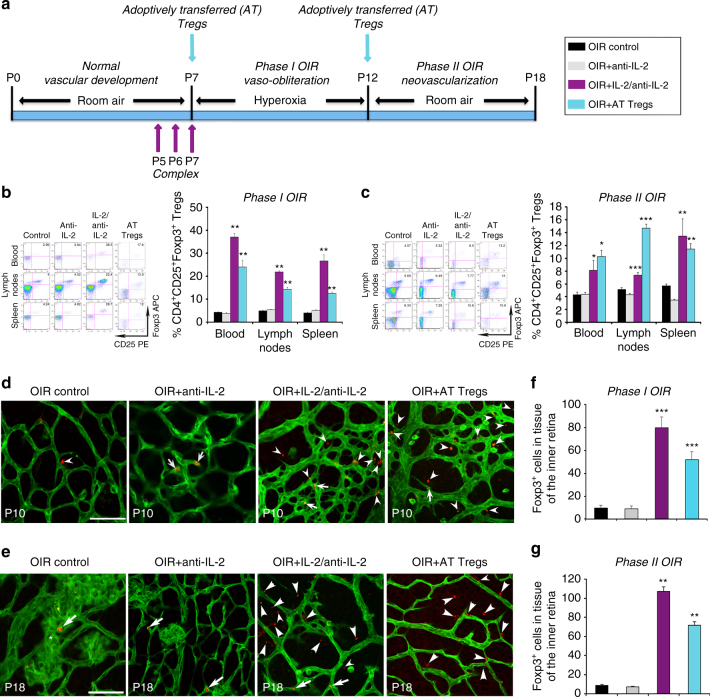
Expanding Treg numbers increased their abundance in OIR and retina. **a** C57BL/6J mice were administered an IL-2/anti-IL-2 mAb complex at postnatal day (*P*) 5, P6, and P7. Separate cohorts of OIR mice received purified splenic Tregs by adoptive transfer (AT) at P7 and P12. Mice were studied in phase I OIR and phase II OIR and comparisons made to age-matched OIR control mice or OIR mice administered an anti-IL-2 control mAb. FACS revealed that in phase I OIR at P12 **b** and phase II OIR at P18 **c** the numbers of Tregs in blood, pooled lymph nodes, and spleen were increased with both treatments (enlarged FACS plots in Supplementary Fig. 3). Fluorochromes are phycoerythrin PE) and allophycocyanin (APC). *P < 0.05, **P < 0.01, and ***P < 0.001 to OIR controls and OIR + anti-IL-2 mAb controls (one-way ANOVA with Mann–Whitney *U* test). *n = *6 mice/OIR control and OIR + anti-IL-2 group and 7 mice/OIR-treated group at P12 and P18. Representative confocal images of flat mounted retinas from Foxp3^rfp^ mice labeled with isolectin (*green*) to show blood vessels. In phase I OIR at P10 **d** and phase II OIR at P18 **e**, few Foxp3^+^ cells (*red*) were present in retinal tissue and (*arrowhead*) and blood vessels (*arrows*). *Asterisk* denotes neovascular tuft. In OIR-treated groups, Foxp3^+^ cells were increased in retinal tissue (*arrowheads*). *Scale bar*, 50 μm. **f**, **g** Foxp3^+^ cells in tissue of the inner retina (one-way ANOVA with Mann–Whitney *U* test). **P < 0.01 and ***P < 0.001 to OIR control groups. *n* = 13 mice/OIR control group and 9 mice/OIR-treated group at P10. *n* = 12 OIR mice, 9 OIR + anti-IL-2 mice and 7 mice/OIR-treated groups at P18. Values are expressed as mean ± s.e.m

We conducted a ‘proof-of-principle’ study to determine if increasing Treg numbers would elevate their abundance in the retina and prevent their decline at P18 during OIR. Two approaches were employed; first, using an IL-2/anti-IL-2 mAb complex (JES6-1 IL-2 mAb) to expand Treg numbers in vivo^33^, and second, by performing the adoptive transfer of purified Tregs^27^ (Fig. 2a). The extent of retinal vaso-obliteration that occurs in phase I OIR at P12 predicts the degree of compensatory neovascularization that occurs in phase II OIR at P18^34^. In light of this, the treatments were administered to C57BL/6J mice prior to and during phase I OIR and the frequency of Tregs in blood, lymphoid organs, and retina was measured at P12 and in separate cohorts of C57BL/6J mice at P18. In OIR at P12, the IL-2/anti-IL-2 mAb complex increased Treg numbers by 10.1-fold in blood, 4.1-fold in pooled lymph nodes, and 5.3-fold in spleen compared to OIR untreated and OIR anti-IL-2 mAb controls (Fig. 2b and Supplementary Fig. 3). In phase II OIR at P18, Treg numbers had declined, but remained elevated by 1.9-fold in blood, 1.4-fold in pooled lymph nodes, and 2.4-fold in spleen compared to OIR control groups (Fig. 2c and Supplementary Fig. 3). In separate cohorts of mice, Tregs were isolated from Foxp3^rfp^ mice and adoptively transferred into C57BL/6J OIR recipient mice. Treg numbers in blood and lymphoid organs were augmented to a similar extent as seen with the IL-2/anti-IL-2 mAb complex (Fig. 2b, c). The treatments did not influence the body weights of OIR mice (Supplementary Table 1) and predominantly increased Treg numbers. Indeed, 5 days after treatment when the IL-2/anti-IL-2 mAb complex has been reported to increase the number of Tregs to maximal levels^33^ (P10 in our study), the treatments had minimal or no effect on CD8^+^ T cells, natural killer cells, and natural killer T cells (Supplementary Figs. 4 and 5), data that are consistent with previous studies^33^.

An important next step was to determine if the treatment approaches led to the increased penetrance of Tregs into retinal tissue. We evaluated the number of Foxp3^+^ cells in tissue of the inner retina (exterior to blood vessels) of mice with OIR. Both, the IL-2/anti-IL-2 mAb complex and the adoptive transfer of Tregs increased the number of Foxp3^+^ cells in phase I OIR (Fig. 2d, f) and phase II OIR (Fig. 2e, g and Supplementary Fig. 6).

### Expanding Treg numbers reduced retinal vaso-obliteration

**Fig. 3:**
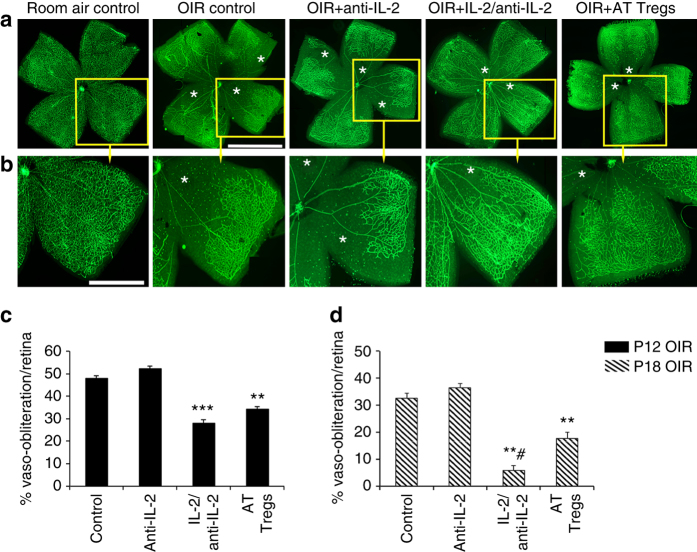
Expanding Treg numbers reduced retinal vaso-obliteration in OIR. **a** Representative confocal images of flat mounted retina from C57BL/6J mice at postnatal day (P) 12 in phase I OIR and labeled with isolectin (*green*) to show blood vessels. *Scale bar*, 0.5 mm. **b** One quadrant (*yellow box*) from each retina has been enlarged. *Scale bar*, 0.25 mm. Retinas from room air control mice show normal vascularization, while retinas from OIR controls and OIR + anti-IL-2 mAb have vaso-obliterated blood vessels (*asterisks*) in the central retina. Mice treated with an IL-2/anti-IL-2 mAb complex or adoptively transferred (AT) Tregs have reduced retinal vaso-obliteration. Quantitation in phase I OIR at P12 **c** and phase II OIR at P18 **d** **P < 0.01 and ***P < 0.001 to OIR control groups. ^#^ *P < *0.05 to OIR + AT Tregs (one-way ANOVA with Mann–Whitney *U* test). *n* = 9 mice/group with three litters examined. Values are mean ± s.e.m

Based on these findings, we speculated that increasing Treg numbers might influence retinal vasculopathy. We examined retina from mice in phase I OIR and found that vaso-obliteration was not altered by the anti-IL-2 control mAb, but was reduced with the IL-2/anti-IL-2 mAb complex or adoptively transferred Tregs (Fig. 3a–c). Although retinal vaso-obliteration in OIR is maximal at P12, it persists until P18. The IL-2/anti-IL-2 mAb complex and adoptively transferred Tregs reduced retinal vaso-obliteration in OIR retina at the P18 time point, as reflected by the increased density of blood vessels in the central retina (Fig. 3d). Separate cohorts of OIR mice served as additional controls by receiving effector T cells (CD4^+^ Foxp3^−^), which had no effect on retinal vaso-obliteration (Supplementary Fig. 7).

### Expanding Treg numbers reduced retinal neovascularization

**Fig. 4:**
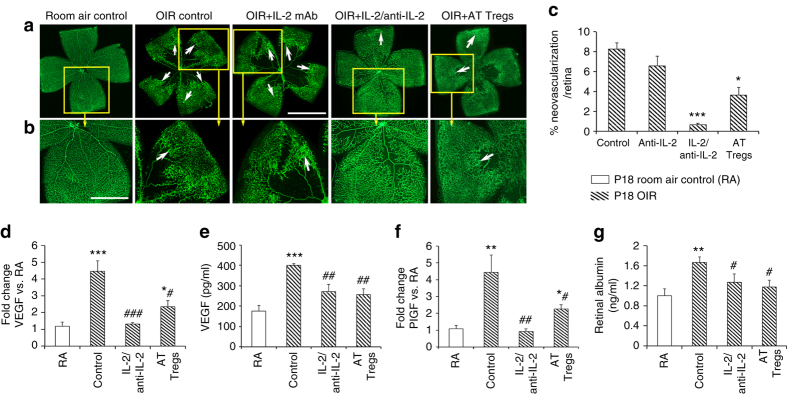
Expanding Treg numbers reduced retinal neovascularization in OIR. **a** Representative confocal images of flat mounted retina from C57BL/6J mice at postnatal day (*P*) 18 in phase II OIR and labeled with isolectin to show blood vessels (*green*). *Scale bar*, 0.5 mm. **b** One quadrant (*yellow box*) from each retina has been enlarged. *Scale bar*, 0.25 mm. Retinas from room air control (RA) mice exhibit normal vascularization, while retinas from OIR controls and OIR + anti-IL-2 mAb display marked neovascularization (*arrows*). OIR mice treated with an IL-2/anti-IL-2 mAb complex or adoptively transferred (AT) Tregs have reduced retinal neovascularization. **c** Neovascularization is expressed as a percentage of total retinal area. *P < 0.05, ***P < 0.001 to OIR control groups (one-way ANOVA with Mann–Whitney *U* test). *n* = 7 room air control mice and 9 mice/OIR group with three litters examined. **d** *vegfa* mRNA, **e** VEGF protein, **f** placental growth factor mRNA, and **g** vascular leakage were increased in retina from OIR mice compared to RA, and in OIR were reduced by both treatments. *P < 0.05, **P < 0.01, and ***P < 0.001 to RA. ^#^ *P < *0.05, ^##^ *P < *0.01, and ^###^ *P < *0.001 to OIR control (one-way ANOVA with Mann–Whitney *U* test). *n* = 5 room air control mice and 7 mice/OIR group. Values are expressed as mean ± s.e.m

We next evaluated the effect of the treatments on the retinal neovascularization and vascular leakage that occurs at P18 in OIR. We found that both the IL-2/anti-IL-2 mAb complex and adoptively transferred Tregs markedly reduced retinal neovascularization, with the former treatment the most effective (Fig. 4a, b). We assessed if these treatments influenced the expression of key pro-angiogenic factors. As the anti-IL-2 control mAb had no influence on retinal vaso-obliteration and neovascularization, we excluded this group from subsequent analyses. We found that in OIR both treatments reduced the expression of VEGF (Fig. 4c, d) and placental growth factor (Fig. 4e) as well as vision-threatening vascular leakage as reflected by reduced retinal albumin permeability (Fig. 4f).

### Expanding Treg numbers altered microglia activation

**Fig. 5:**
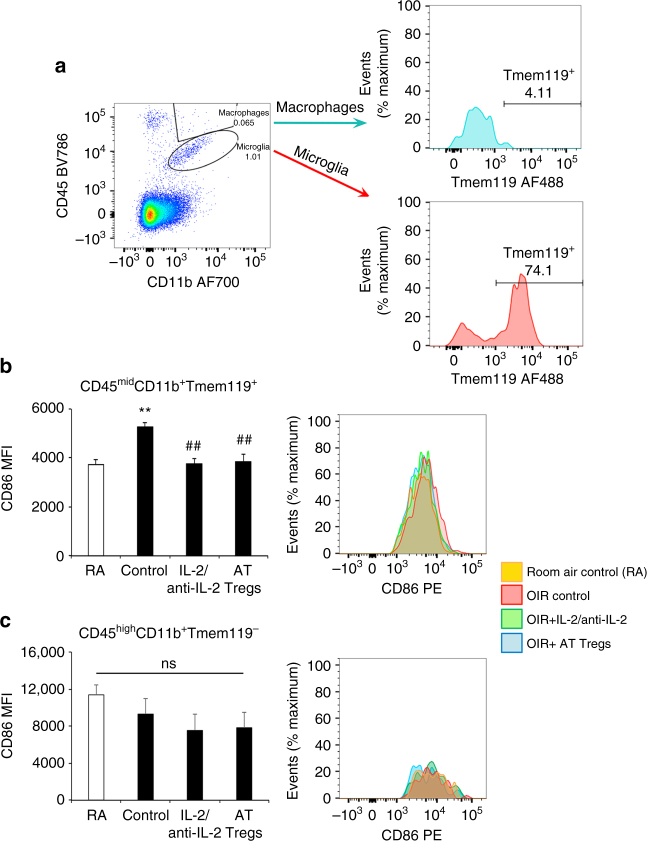
Expanding Treg numbers altered the activation state of microglia. Flow cytometry of retina from C57BL/6J mice at postnatal day 10 in phase I OIR. Fluorochromes are AF700, AF488 and phycoerythrin (PE). **a** FACS plot schema showing cell populations in OIR retina revealed by CD45 and CD11b staining. Microglia (CD45^mid^CD11b^+^) rather than macrophages (CD45^high^CD11b^+^) predominate and Tmem119 is restricted to CD45^mid^CD11b^+^ microglia. **b** Mean fluorescence intensity (MFI) of CD86 in CD45^mid^CD11b^+^Tmem119^+^ microglia showing an increase with OIR compared to room air controls (RA), and a reduction in OIR mice following treatment with the IL-2/anti-IL-2 mAb complex and adoptively transferred (AT) Tregs. **c** MFI of CD86 in CD45^high^CD11b^+^Tmem119^−^ macrophages showing no change with OIR compared to RA or the treatments. **P < 0.01 to RA. ^##^ *P < *0.01 to OIR control (one-way ANOVA with Mann–Whitney *U* test). NS, not significant. *n = *5 mice group. Values are expressed as mean ± s.e.m

**Fig. 6:**
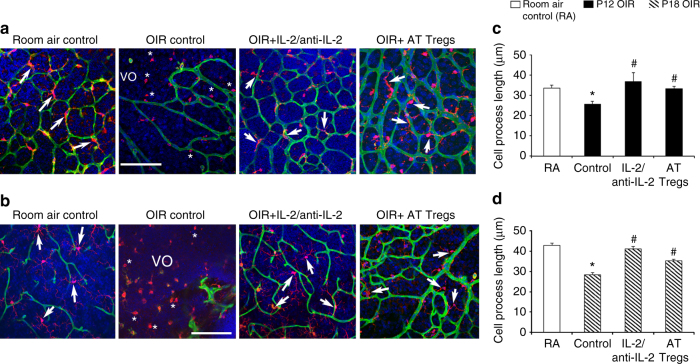
Expanding Treg numbers preserved the ramified phenotype of microglia. Representative confocal images of flat mounted retina from C57BL/6J mice showing blood vessels (isolectin, *green*), microglia (Iba1^+^, *red*), and nuclei (DAPI, *blue*). *Scale bar*, 100 μm. In room air controls (RA) at **a** P12 and **b** P18, Iba1^+^ cells with long ramified processes (*arrows*) predominate, while in OIR at both time points, most Iba1^+^ cells exhibit short cell processes (*asterisks*) and are found in the vaso-obliterated (*VO*) retina. In OIR mice treated with the IL-2/anti-IL-2 mAb complex or the adoptive transfer (AT) of Tregs, the number of Iba1^+^ cells with long ramified cell processes (*arrows*) predominate. The primary cell process of each Iba1^+^ cell per field of retina was quantitated at **c** P12 and **d** P18. *P < 0.05 to RA. ^#^ *P < *0.05 to OIR control (one-way ANOVA with Mann–Whitney *U* test). *n = *6 room air control mice and 8 mice/OIR group. Values are expressed as mean ± s.e.m

To determine if expanded Treg numbers influenced the activation state of microglia and macrophages, we examined phase I OIR. Macrophages^35^ are commonly identified by their expression of the common leukocyte antigen CD45^high^ and microglia^35^ as CD45^low^. By using flow cytometry to evaluate the expression of CD45 as well as CD11b, we determined that in the OIR retina, CD45^high^CD11b^+^ macrophages comprise only 0.05% of the total number of retinal cells and that CD45^mid^CD11b^+^ microglia predominate (Fig. 5a). We confirmed that CD45^mid^CD11b^+^ cells were microglia, by examining the expression of the transmembrane protein, Tmem119, which is exclusively expressed by microglia and not myeloid cells in the central nervous system^18^. Microglia were identified as CD45^mid^CD11b^+^Tmem119^+^ and the co-stimulatory molecule CD86 was used as a marker of activation and responsiveness to Tregs^19, 20^. Indeed, CD86 expression in CD45^mid^CD11b^+^Tmem119^+^ microglia was increased in OIR retina when compared to room air controls, and in OIR mice was reduced following treatment with either the IL-2/anti-IL-2 mAb complex or adoptively transferred Tregs (Fig. 5b). In contrast, CD86 expression in CD45^high^CD11b^+^Tmem119^−^ macrophages in retina was unaffected by OIR or the treatments (Fig. 5c). To evaluate the phenotype of these cells, we performed confocal microscopy with ionized calcium-binding adapter molecule-1 (Iba1) and quantitated cell process length as microglia when resting have ramified cell processes and when activated have shorter cell processes^13^. In phase I and phase II of OIR, Iba1^+^ cells had shorter processes than in room air controls (Fig. 6a, b). Furthermore, the number of Iba1^+^ cells with long cell processes increased following treatment with the IL-2/anti-IL-2 mAb complex or adoptively transferred Tregs (Fig. 6c, d).

### In vitro Tregs altered the activation of microglia

**Fig. 7:**
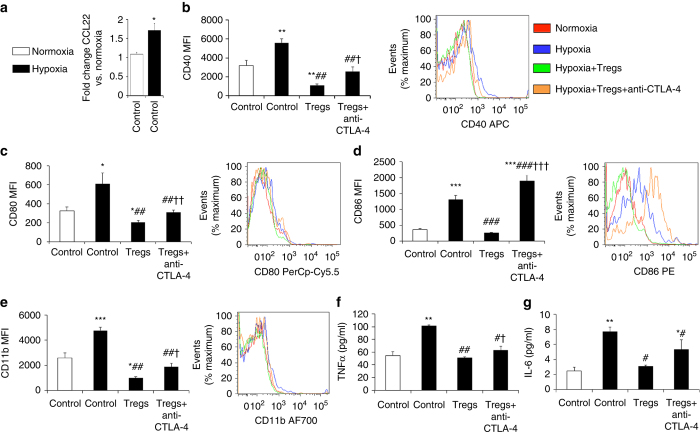
In vitro Tregs alter the activation of retinal microglia. **a** Primary cultures of microglia were established from the retinas of 10 to 12-day-old C57BL/6J mice. Quantitative real-time PCR revealed that *ccl22* mRNA levels were increased by hypoxia (0.5% O_2_). *P < 0.05. **b**–**g** Retinal microglia were exposed to hypoxia (0.5% O_2_) and co-cultured with Tregs and a blocking anti-CTLA-4 mAb (10 μg/ml). Flow cytometry was performed and data are expressed as mean fluorescence intensity (*MFI*) for **b** CD40, **c** CD80, **d** CD86, and **e** CD11b. Fluorochromes are allophycocyanin (*APC*), PerCp-Cy5.5, phycoerythrin (*PE*), and AF700. *P < 0.05, **P < 0.01, and ***P < 0.001 to normoxia control. ^##^ *P < *0.01 and ^###^ *P* < 0.001 to hypoxia control. ^†^ *P < *0.05, ^††^ *P < *0.01, and ^†††^ *P < *0.001 to hypoxia + Tregs. In the cell supernatant, an ELISA was used to measure **f** TNFα and **g** IL-6 levels in cell supernatant. *P < 0.05 and **P < 0.01 to normoxia control. ^#^ *P < *0.05 and ^##^ *P < *0.01 to hypoxia control. ^†^ *P < *0.05 to hypoxia + Tregs. Data are representative of two independent experiments each containing three replicates (one-way ANOVA with Student’s *t* test). Values are expressed as mean ± s.e.m

To determine if retinal microglia have the ability to attract Tregs to the retina, we exposed primary cultures established from neonatal retina to hypoxia^16^, an in vitro counterpart of the ischemia-induced microglial activation that occurs in phase II of OIR. As our flow cytometry studies of mouse retina demonstrated that macrophages constitute an extremely small proportion of the total pool of microglial and macrophage cells, we refer to these cultures as microglia. Focusing on CCL22, a chemokine expressed by activated macrophages that promote Treg migration^36^, we found *ccl22* mRNA levels in microglia increased in response to hypoxia (Fig. 7a). One mechanism by which Tregs influence macrophage activation is via the release of anti-inflammatory cytokines^21^. We performed transwell experiments involving retinal microglia and Tregs and found no change in the activation state of microglia as assessed by the expression of the co-stimulatory molecules, CD40, CD80, and CD86^23^ as well as CD11b (Supplementary Fig. 8). Tregs can also modulate macrophage activation through cell-to-cell contact^23^. Therefore, we cultured retinal microglia from young mice and activated them by hypoxia. When co-cultured with Tregs obtained from adult mice, CD40, CD80, and CD86 were reduced as well as the activation marker CD11b (Fig. 7b–e). Furthermore, levels of tumor necrosis factor-alpha (TNFα) and IL-6, pro-inflammatory and pro-angiogenic agents in the retina^37, 38^ were also reduced (Fig. 7f, g). A blocking anti-CTLA-4 mAb, which inhibits the suppressive actions of Tregs^19^, partially blunted these responses (Fig. 7b–g).

**Fig. 8:**
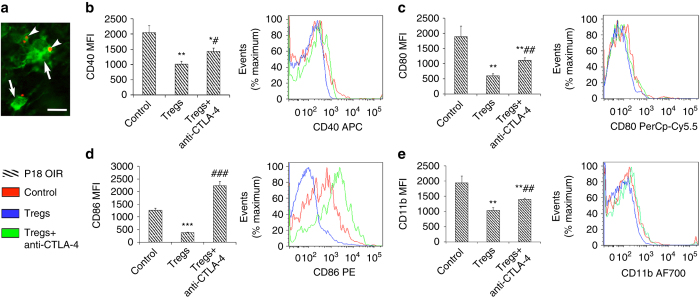
The activation state of OIR microglia is altered by Tregs. **a** Representative confocal image of a flat mounted retina from Foxp3^rfp^ mice at postnatal day (P) 18 with OIR and treated with the IL-2/anti-Il-2 mAb complex. Foxp3^+^ cells (*red*, *arrowheads*) contact microglia (Iba1 immunolabeling in *green*, *arrows*). *Scale bar*, 40 μm. **b**–**e** Microglia from retinas of OIR mice at P18 were co-cultured with Tregs purified from adult mouse spleen and incubated with a blocking anti-CTLA-4 mAb (10 μg/ml). Flow cytometry was performed and mean fluorescence intensity (MFI) quantitated for **b** CD40, **c** CD80, **d** CD86, and **e** CD11b. Fluorochromes are allophycocyanin (*APC*), PerCp-Cy5.5, phycoerythrin (*PE*), and AF700. *P < 0.05, **P < 0.01, and **P < 0.001 to OIR control. ^#^ *P < *0.05, ^##^ *P < *0.01, and ^###^ *P < *0.001 to OIR + Tregs. Data are representative of two independent experiments each containing three replicates (one-way ANOVA with Student’s *t* test). Values are expressed as mean ± s.e.m

Importantly, the ability of Tregs to contact and alter the activation state of retinal microglia was confirmed in vivo in OIR. Treatment with the IL-2/anti-IL-2 mAb complex resulted in Tregs associating with retinal microglia (Fig. 8a). Moreover, microglia obtained from OIR retina and co-cultured with Tregs from the spleens of adult mice resulted in reduced CD40, CD80, CD86, and CD11b (Fig. 8b–e).

## Discussion

The major findings of this study are that we have identified a key role for Tregs in OIR, which is of potential clinical significance as a new treatment approach for this condition. Specifically, our data demonstrate for the first time that the acute retinal ischemia of OIR is a strong but transient stimulus for the expansion of Treg numbers in lymphoid organs. Furthermore in OIR, Tregs migrate to the damaged retina but are insufficient in number to attenuate vaso-obliteration and prevent the advancement of vascular damage to neovascularization and vascular leakage. These unexpected findings opened up the new possibility that harnessing the immunosuppressive capabilities of Tregs is a possible strategy to attenuate vasculopathy in the retina. Indeed, a major finding was that augmentation of the Treg compartment led to increased trafficking of Tregs into the retina and a marked reduction in the hallmark features of sight-threatening neovascular retinopathy; specifically vaso-obliteration, neovascularization, and vascular leakage as well as elevated levels of VEGF, a key mediator of retinal angiogenesis and vascular leakage^2, 39^. These novel findings reveal the substantial protective actions of Treg cell therapy as a potential treatment for neovascular retinopathy.

There is growing evidence that systemic inflammation is involved in the development of retinopathy of prematurity^9, 11^ with affected infants having increased circulating levels of IL-6, IL-8, and TNFα^40^. Moreover, results from a very large cohort study indicate that the levels of circulating pro-inflammatory cytokines are elevated at multiple time points in preterm infants who later develop retinopathy of prematurity^10^. The OIR model allows the inflammatory profile of the retina to be directly interrogated. Indeed, it has been shown that increased amounts of pro-inflammatory and pro-angiogenic cytokines, such as TNFα^41^ and IL-17^16^, are produced in the retina by microglia^16, 41^. The ability of Tregs to suppress inflammation^19^, including macrophage activation^20–22^, albeit such studies had been performed in other disease settings, led us to postulate that a paucity of Treg numbers in OIR may allow inflammation in the retina to proceed unchecked. However, our finding that the number of Tregs in lymphoid organs peaked in the acute stage of retinal ischemia at P13 of phase II OIR, when pro-inflammatory cytokines are elevated in the retina^41^, suggested that a temporary expansion of Tregs might represent a response to dampen this burst of intense local retinal inflammation. The Foxp3^+^ reporter mouse was an important tool to clearly demonstrate that at this time point in OIR, Tregs do indeed migrate to the retina and are not only present in blood vessels but also penetrate retinal tissue in increased numbers. The factors involved in the recruitment of Tregs to the ischemic retina are likely to be multiple. Specifically, we determined that the chemokine CCL22, a strong chemoattractant for Tregs^36^, is involved as its expression was increased in retinal microglia exposed to hypoxia. Other chemokines known to influence the homing of T cells, such as RANTES (regulated on activation, normal T cell expressed and secreted), may also participate, with high levels of this chemoattractant detected in retinal tissue and microglia in OIR^42^ as well as in the vitreous fluid of infants with retinopathy of prematurity^10, 43^. Our finding of an acute increase followed by a decline in the number of Tregs in lymphoid organs and retina is consistent with previous studies of other ischemic diseases such as myocardial infarction^44, 45^, and suggested to us that expanding the Treg compartment might protect the retina against a highly pro-inflammatory environment and the escalating damage to the vasculature that develops in OIR.

We evaluated if two approaches that increase the number of Tregs in other disease models^26, 27, 33^ would be retinoprotective when administered during phase I OIR and result in a reduction in retinal vaso-obliteration at this time point. We viewed the timing of administration of these treatments in phase I OIR to be important as the extent of vaso-obliteration in phase I OIR influences the degree of subsequent neovascularization and vascular leakage in phase II OIR^34^. Therefore, strategies to repair the vaso-obliterated retina or allow the prevention of its occurrence in phase I OIR have the potential to markedly attenuate sight-threatening vasculopathy. Both treatments predominantly and markedly increased the number of Tregs in lymphoid organs and blood in phase I OIR and had a minor effect on other immune cells as reported previously^33^. Although we cannot exclude the possibility that these other immune cell populations influenced OIR, it was clear that both treatments increased the number of Tregs in the inner retina in phase I OIR with Tregs mainly found in retinal tissue, suggesting transmigration from retinal blood vessels. Furthermore, the IL-2/anti-IL-2 mAb complex was extremely effective in reducing retinal vaso-obliteration in phase I OIR and residual vaso-obliteration in phase II OIR. The second approach involving the adoptive transfer of Tregs was similarly effective as the IL-2/anti-IL-2 mAb complex, and has clinical relevance as it is currently being explored in clinical trials for diseases such as type 1 diabetes^28, 46^.

Of particular importance was our finding that the two treatment approaches and the timing of their administration had substantial impact on retinal vasculopathy in phase II OIR. This was evidenced by the sustained elevation of Tregs in lymphoid organs and blood, although this occurred to a lesser degree than in phase I OIR, increased trafficking of Tregs into retinal tissue and a marked reduction in retinal neovascularization and vascular leakage. Furthermore, both approaches reduced the elevated levels of VEGF in the retina, which in conjunction with family members, such as placental growth factor^47^, is an important target for the treatment of patients with neovascular retinopathies^1^. The ability of Tregs to migrate into retinal tissue presumably occurred through the leaky blood vessels in the inner retina that characterize OIR. A limitation of our study is that we did not investigate the method of migration of Tregs into retinal tissue and if Tregs also penetrate the retina through the outer blood–retinal barrier (retinal pigment epithelium and choroid) as reported for monocytes and microglia/macrophages^48, 49^. This is a possibility as it is increasingly appreciated that the retinal pigment epithelium/choroid becomes compromised during phase II OIR and persists in adult mice^50^, a pathology that can also develop in adults with a history of retinopathy of prematurity^51^. Therefore, it is conceivable that we have underestimated the number of Tregs that are present in the retina, particularly in phase II OIR. Nevertheless, together our results highlight the powerful preventative and protective effects of Tregs on the key elements that drive vascular pathology and vision loss in the retina.

To interrogate the mechanisms whereby Tregs attenuate vasculopathy, we focused on microglia due to their central role in retinal vascular disease^13, 16, 17^ and their similarity to macrophages, as well as the capacity of Tregs to influence macrophage activation^20–22^. Until recently, methods to distinguish microglia from macrophages have been limited. Only in the last year have Bennett and colleagues demonstrated that the type 1A single-pass transmembrane protein Tmem119 distinguishes microglia from resident and infiltrating macrophages as well as other immune cells and neural cell types in healthy and diseased brain from mice and humans^18^. To our knowledge, our finding that CD45^mid^CD11b^+^Tmem119^+^ microglia predominate over macrophages in the retina in the early stages of OIR has not previously been demonstrated. Tmem119 is developmentally regulated^18^, which may explain why 74% of microglia expressed Tmem119 in the neonatal retina in phase I OIR. These microglia are in a heightened state of activation and responsiveness to Tregs as shown by their increased expression of CD86, a co-stimulatory molecule that has a key role in interacting with Tregs^19, 20^. Our finding that both treatments altered the activation state of microglia but not Tmem119^−^ macrophages, indicate a likely interaction between microglia and Tregs in the OIR retina. These results are consistent with our phenotypic evaluation of retinal microglia, which we found to largely be in a ramified and presumably resting state in OIR following treatment with either the IL-2/anti-IL-2 complex or the adoptive transfer of Tregs.

It is possible that the altered activation state of retinal microglia was not a direct consequence of the increased number of Tregs. Hence, our co-culture experiments were performed to confirm that indeed this is a direct effect. Treg-mediated immunosuppression can occur via their release of soluble factors as well as by cell-to-cell contact^19–21^. Transwell experiments revealed that any soluble factors released from Tregs did not influence the activation state of retinal microglia and their responsiveness to Tregs, as there was no change in the expression of the co-stimulatory molecules CD40, CD80, and CD86. Nevertheless, it is possible that in vivo, soluble factors such as IL-10, IL-4, and transforming growth factor-β cannot be excluded. In our studies, cell-to-cell contact appears to be the primary mechanism by which Tregs influence the activation state of retinal microglia exposed to hypoxic culture conditions, as well as those from the diseased OIR retina via a reduction in CD40, CD80, and CD86. Re-inforcing our findings was the reversal of these events by blockade of CTLA-4, a protein that promotes the immunosuppressive capabilities of Foxp3^+^ Tregs^19^. Complete reversal did not occur, which is consistent with evidence that other signaling molecules are also involved in regulating the immunosuppressive function of Tregs^52^. We acknowledge that these findings should be interpreted in the context that potentially retinal microglia in vitro might have different characteristics to retinal microglia in vivo, and that retinal macrophages, albeit present in small numbers in our studies, may also be responsive to Tregs. Further, we do not exclude the possibility that in OIR, microglia are not the primary mechanism by which Tregs reduce retinal vasculopathy, but that Tregs might influence the pro-inflammatory status and health of various cell types in the retina, an area of interest that could reveal further valuable information about the role of Tregs in neovascular retinopathy. Nevertheless, collectively our data provide compelling evidence for a direct interaction between Tregs and retinal microglia that results in the reduction of potent pro-inflammatory and pro-angiogenic factors in the retina such as TNFα and IL-6^37, 38^.

In summary, we have demonstrated that Tregs penetrate the ischemic retina and when amplified are able to alter the activation state of microglia and reduce severe microvascular disease. Finally, our work demonstrates that maximizing the immunosuppressive capacity of Tregs has the potential to be an effective new addition to current inadequate treatments for blinding neovascular retinopathies, such as retinopathy of prematurity, diabetic retinopathy, and age-related macular degeneration.

## Methods

### Animals and treatments

All studies were conducted according to the National Health and Medical Research Council of Australia’s Guidelines for the Care of Animals in Scientific Research, and were approved by the Alfred Medical Research and Education Precinct (AMREP) animal ethics committee. C57BL/6J mice (JAX stock number 000664) were purchased from the Animal Resources Centre (Perth, Western Australia). *Foxp3* ^*rfp*^ mice on a C57BL/6J background were obtained from Professor Richard A. Flavell (Howard Hughes Medical Institute, Yale University School of Medicine, New Hampshire, CT, USA)^32^. All mice were housed in AMREP Animal Services (Melbourne, VIC, Australia). Litters of mice were randomized to OIR or room air control groups as well as treatment groups. In OIR studies, both male and female mouse pups and their mothers were exposed to hyperoxia (75% O_2_) for 21 h per day between postnatal day (P) 7 to P12 in specialized chambers that were maintained by a Proox 110 gas regulator (BioSpherix, NY, USA) and 10 m^3^ medical oxygen cylinders (Air Liquide Australia, VIC, Australia). Mice were subsequently exposed to room air until P18^16, 31^ (Fig. 1a). Age-matched controls were housed in room air. Separate groups of male and female mouse pups were studied at P10, P12, P13, P15, and P18. To expand Foxp3^+^ Tregs, mice were administered an IL-2/anti-IL-2 mAb complex^33^ at P5, P6, and P7. 1.5 µg each of recombinant mouse IL-2 (R&D Systems, MN, USA) and purified anti-mouse IL-2 (JES6-1 IL-2 mAb Biolegend, San Diego, CA, USA, item number 503702) were incubated for 30 min at 37 °C to form a complex before administering via a single intraperitoneal injection (Fig. 2a). Comparisons were made to mice administered an IL-2 mAb control at the same time points. However, off-target effects from unspecific activation of Fc receptors by the IL-2/anti-IL-2 mAb complex cannot be excluded using this control. In studies involving the administration of Tregs by adoptive transfer, Tregs were enriched from lymphocytes obtained from Foxp3^rfp^ mice, which were sorted using a FACS CBD Influx (BD Biosciences, San Jose, CA), and purity was >95% CD4^+^Foxp3^+^ cells. C57BL/6J mice were randomized to receive 1 × 10^6^ Tregs by a single intraperitoneal injection at P7 and then killed at P12. Separate groups of male and female C57BL/6J mouse pups received 1 × 10^6^ Foxp3^+^ Tregs at P7 and again at P12, and mice were killed at P18 (Fig. 2a). Controls received effector T cells (CD4^+^ Foxp3^−^).

OIR mice were only included in the study if they consistently gained body weight reaching ~5 g by P10, 5.5 g by P12–P13 and 7 g by P18 (Supplementary Table 1) in accordance with established criteria for studies of OIR^53^. Mice that did not achieve these body weights were not included in any analyses. Mice were killed with sodium pentobarbitone (170 mg/ml, Virbac, Peakhurst, NSW, Australia).

### Flow cytometry

The retina, spleen, and lymph nodes (pooled inguinal, auxillary, brachial, deep cervical, and superficial cervical) were excised in Hank’s Balanced Salt Solution (HBSS) and 2% heat inactivated fetal bovine serum. Blood from the heart was collected into heparinized syringes using 26G needles. Spleen and lymph nodes were mechanically dissociated and passed through a 40 µm strainer. Red blood cells were lysed by 1–5 mls of 1× RBC lysis buffer (eBioscience) for 2–5 min at room temperature. Retina was digested in collagenase D (1 mg/ml) and DNase I (15 U/ml) in RPMI 1640 for 45 min at 37 °C. The homogenized tissue was filtered through a 70 µm strainer (BD Biosciences) and quenched with FACS buffer (phosphate buffered saline, PBS, with 1% BSA and 0.5 mM EDTA). Cells from lymphoid tissues and blood were then exposed to Fc Block (BD Biosciences) and incubated in FACS buffer containing 1:500 rat anti-mouse CD3e (145-2C11, BD Bioscience, item number 563062), 1:2000 CD4 (RM4-5, BD Biosciences, item number 550954), 1:1000 CD8a (53-6.7, BD Bioscience, item number 563046), 1:500 CD25 (7D4; BD Biosciences, item number 553866), and 1:500 NK1.1 (PK136, BD Bioscience, item number 550627). Cells were further processed with a Fixation/Permeabilization Kit according to the manufacturer’s instructions (eBioscience, CA, USA) before incubation with 1:500 Foxp3 (FJK-16s; eBioscience, item numbers 61-5773-82 and 17-5773-82) for 45 min at 4 °C. Retinal cells were incubated in blocking buffer (1% goat serum in 0.1 M PBS) and incubated with 1:100 Tmem119 antibodies^18^ or 15 min at room temperature with gentle rotation. Retinal cells were washed twice with FACS buffer and incubated with a goat anti-rabbit IgG Alexa Fluor-488 antibody (1:500, A-11008, ThermoFisher, item number A-11008). Cells were washed with FACS buffer and further incubated in FACS buffer containing the following antibodies: 1:500 CD45 (30-F11; BD Biosciences, item number 564225), 1:200 CD11b (M1/70; BD Biosciences, item number 557960), and 1:1000 CD86 (GL-1, eBioscience, item number 561963) for 45 min at 4 °C. FACS analysis was performed on a BD LSR II flow cytometer (BD Biosciences). Dead cells were excluded through staining with either propidium iodide or Invitrogen LIVE/DEAD Fixable Aqua (Life Technologies, VIC, Australia). A minimum of 100,000 events were collected per sample and analyzed with FlowJo software (Tree Star, Ashland, OR, USA).

### Microglial cell processes

Eyes were fixed in 4% paraformaldehyde for 30 min and retinal dissection was performed in a Petri dish filled with PBS under a dissecting microscope. Each retina was detached from the choroid using forceps and four radial cuts extending about 2/3 from the edge of the retina were made to obtain retinal flat mounts. For immunohistochemistry, investigators were blinded to the experimental groups. Retina were first incubated for 1 h at room temperature with 10% normal goat serum (NGS) in 0.3% Triton-100 at room temperature, and then overnight at 4 °C with anti-mouse Iba1 (1:100, Wako, Tokyo, Japan, item number 019-197410) dissolved in 1% NGS, 0.3% Triton X-100, and PBS. Six successive 10 min washes were performed with wash buffer (0.3% Triton X-100 in PBS). Retina was then incubated for 1.5 h at room temperature with anti-rabbit IgG Alexa Fluor^®^ 548 (Molecular Probes, Eugene, OR, USA) dissolved in 1% NGS and 0.3% Triton X-100. This was followed by six washes, and subsequent incubation with FITC-conjugated isolectin (1:100, L5264, Sigma, St. Louis, MI, USA) in 1% Triton X-100. After washing, retina was counterstained with 4′,6-diamidino-2-phenylindole (DAPI, 300 nM, Molecular Probes) and mounted in fluorescent mounting medium (DakoCytomation, Glostrup, Denmark). Retinal wholemounts were visualized with a Nikon A1 laser scanning confocal microscope (Nikon Instruments Inc., Melville, NY, USA), with a ×40 water immersion objective lens. Images were captured with Nikon image software (NIS-Elements AR 3.0). Ten fields were randomly selected per retina from the ganglion cell layer to the inner plexiform layer. Cell process length was measured from the center of the microglia soma to the process tip using ImageJ software (v3.1, National Institutes of Health, Bethesda, Washington). The primary cell process of each microglia was measured.

### Retinal vaso-obliteration and neovascularization

Retinal flat mounts were stained with FITC-conjugated isolectin as described above and individual retinal images were acquired at ×100 magnification using a digital microscope camera (AxioCam MRc 6.1.0.0, Carl Zeiss, Gottingen, Germany) attached to a Zeiss Axio ×1 microscope (Carl Zeiss). Entire retinal montages were achieved using the tiling tool in the AxioObserver software (v5.3, Carl Zeiss). ImageJ was used to quantitate vaso-obliteration using the freehand tool, and neovascularization by psuedocolorizing aggregates of blood vessels^16, 31^. For room air controls, seven mice (males and females) were evaluated and for OIR groups, nine mice (males and females) per group were studied from three different litters of mice at P12 and P18. Investigators were blinded to the identity of the experimental groups.

### Quantitation of Foxp3^+^ cells in retinal wholemounts

Retinal flat mounts were prepared from Foxp3^rfp^ mice and stained with FITC-conjugated isolectin as described above and Foxp3^+^ cells were separately counted in retinal blood vessels and exterior to blood vessels in retinal tissue. The inner region of the retina (inner limiting membrane, ganglion cell layer, inner plexiform layer, inner nuclear layer, outer plexiform layer) was evaluated.

### Quantitative PCR

We utilized a previous method^16, 31^, whereby total RNA was isolated from single retina using the RNeasy mini kit (Qiagen, Doncaster, VIC, Australia), and then 1 µg of RNA was subjected to DNase treatment (DNA-free kit, Ambion, Carlsbad, CA, USA) and reverse transcription (First Strand cDNA synthesis kit, Roche, Switzerland). mRNA expression was normalized to 18 s rRNA endogenous control and the relative fold difference in expression was calculated using the comparative 2^−ΔΔCt^ method. The primer sequences for *vegfa* ^16^ are, forward primer: 5′-AGCAGAAGTCCCATGAAGTGATC-3′ and reverse primer: 5′-TCAATCGGACGGCAGTAGCT-3′. The primer sequences for placental growth factor are forward primer: 5′-CTGCTGGGAACAACTCAACAGA-3′ and reverse primer: 5′-GCTGCGACCCCACACTTC-3′. The primer sequences for CCL22 are forward primer: 5′-CACTTCAGACCTCCGATGCA-3′ and reverse primer: 5′-TCCTGGCAGCAGATACTGTCTTC-3′.

### Cell culture

Microglia were obtained from retina of 10–12-day-old mice and established as primary cultures^16^. Retina was maintained in low-glucose Dulbecco’s modified eagle media (DMEM)/2 mM L-glutamine/100U penicillin/100 μg streptomycin/0.25 μg amphotericin B on ice. Retina was then washed with Hanks balanced salt solution (HBSS, without Ca^2+^/Mg^2+^), spun at 1000 × rpm for 5 min, and re-suspended in 0.05% tryspin/0.53 mM ethylenediaminetetracetic acid (EDTA, without Ca^2+^/Mg^2+^) and incubated for 1 h at 37 °C/5% CO_2_, with shaking every 10 min. Digestion was quenched with low-glucose DMEM/10% fetal bovine serum (FBS)/100U penicillin/100 μg streptomycin/0.25 mg amphotericin B. The suspension was then spun at 1000 × rpm for 5 min and the pellet re-suspended in culture medium (low-glucose DMEM, 10% FBS, 100 U penicillin, 100 μg streptomycin, 0.25 μg amphotericin supplemented with +1 ng/ml mouse granulocyte macrophage colony stimulating factor and plated onto T75 flasks coated with 10 μg/ml poly-D-lysine. Cells were cultured until reaching confluence and flasks shaken for 2 h at 200 × rpm at 37 °C to separate microglia from supporting glial cells. After shaking, the culture supernatant was removed, spun down at 1000 × rpm for 5 min, re-suspended in culture medium, and plated onto poly-D-lysine-coated plates or coverslips. Characterization of the cultured microglia was performed with immunocytochemistry for the microglial marker, Iba1. Cells were washed twice with HBSS (without Ca^2+^/Mg^2+^) and fixed with 3.7% formaldehyde in HBSS (without Ca^2+^/Mg^2+^) at 4°C for 10 min. Cells were washed as above and permeabilized with 0.1% Triton X-100 in HBSS (without Ca^2+^/Mg^2+^) for 5 min. After washing as above, cells were blocked with 3% BSA/3% goat serum in HBSS (without Ca^2+^/Mg^2+^) for 15 min. After washing, 2 μg/ml rabbit anti-Iba1 in 3% bovine serum albumin (BSA)/3% goat serum in HBSS (without Ca^2+^/Mg^2+^) was applied and incubated for 2 h. Cells were washed, then incubated with 1:400 goat anti-rabbit IgG-Alexa568 (Invitrogen) in 3% BSA/3% goat serum in HBSS (without Ca^2+^/Mg^2+^) and incubated for 1 h. Cells were washed and stained with 4′,6′-diamino 2-phenylindole (DAPI, Invitrogen, VIC, Australia) at 300 nM in HBSS (without Ca^2+^/Mg^2+^) for 1 min, washed again, and mounted using Dako fluorescent mounting medium (Dako) onto slides. Three fields were counted for Iba1-positive cells in duplicate slides. The purity of microglia was more than 90% determined by Iba1 labeling.

Foxp3^+^ Tregs were isolated from the spleens of adult Foxp3^rfp^ mice as described above. Retinal microglia (5 × 10^4^ cells/well) and Foxp3^+^ Tregs (2.5 × 10^4^ cells/well) were then seeded into 96-well plates in RPMI containing 10% FCS, 1% penicillin/streptomycin, and 0.5 μM 2-ME, and co-cultured for 48 h under normoxia (21% O_2_) or hypoxia (0.5% O_2_) conditions^16^ and with or without a blocking anti-CTLA-4 mAb (10 μg/ml, clone 9H10, Biolegend, USA, item number 106250). In a separate experiment, microglia were purified from the retinas of mice with OIR at P18 mice using CD11b^+^ (microglia) microbeads (purity >95%, Miltenyi Biotech, USA) according to the manufacturer’s instructions. Cells were collected and analyzed for CD11b (clone M1/70, BD Biosciences, item number 557960), CD40 (clone HM10-3, eBiociences, item number 17-1541-82), CD80 (clone 16-10A1, BD Biosciences, item number 560526), and CD86 (clone GL-1, BD Biosciences, item number 561963) by flow cytometry. These microglia from OIR retina were then co-cultured with Foxp3^+^ Tregs obtained from adult Foxp3^rfp^ mice as described above. For transwell experiments, Tregs were plated in a transwell insert (0.4 µm pore size, Corning). Each experiment contained two pooled retina co-cultured with Tregs resulting in six biological replicates.

### ELISA

We used an established protocol to measure VEGF levels and vascular leakage^16^. Retinas were homogenized on ice in 0.01 M sodium phosphate buffer (pH 9.5) containing protease/phosphatase inhibitor cocktail (1:100, Sigma). The total protein concentration was quantitated using a colormetric assay (Biorad, CA, USA). Undiluted retinal lysates were assayed in duplicate using ELISA kits for rat VEGF (#DY493, R&D systems, item number DY493). Albumin levels in retina were measured using a mouse albumin ELISA kit (Bethyl Laboratories, TX, USA, item number E90-134) and normalized to dry retinal weight.

### Cytokine array

Fifty microliter of cell culture supernatant was analyzed according to the BD Cytometric Bead Array Mouse Inflammation Kit (#552364, BD Biosciences) and the manufacturer’s protocol. Results were analyzed using FCAP Array Version 2.0 software (Soft Flow, St. Louis Park, MN, USA).

### Statistics

Data were first assessed for normality by Kolmogorov–Smirnov, D’Agostinos and Pearson ormnibus, as well as Shapiro–Wilk normality tests (GraphPad Prism, San Diego, CA, USA). Analysis was then performed by one-way ANOVA followed by Bonferroni pro-test analysis (for data that passed normality tests) or by nonparametric Kruskal–Wallis test followed by Dunn’s post-test (for data that did not pass normality tests). For comparison between two groups, a one-way ANOVA was followed by either a Student’s *t* test or a Mann–Whitney *U* test on data that passed and failed normality tests, respectively. The sample size was estimated by a power analysis assuming a normal distribution. *P* values smaller than 0.05 was considered to be significant. Investigators were masked to the experimental groups. Values are expressed as mean ± s.e.m.

### Data availability

The authors declare that all data supporting the findings of this study are available within the paper and its Supplementary Information.

## Electronic supplementary material

Supplementary Information
