# Supplementary material for: Foxp3+ Tregs are recruited to the retina to repair pathological angiogenesis
Source: Nat Commun. 2017 Sep 29;8:748. doi: 10.1038/s41467-017-00751-w (PMC5622066; doi:10.1038/s41467-017-00751-w)
Supplement: Supplementary file 1 — Supplementary Information [file 41467_2017_751_MOESM1_ESM.pdf]

File Name: Supplementary Information
Descriptions: Supplementary Figures and Supplementary Table

SUPPLEMENTARY INFORMATION

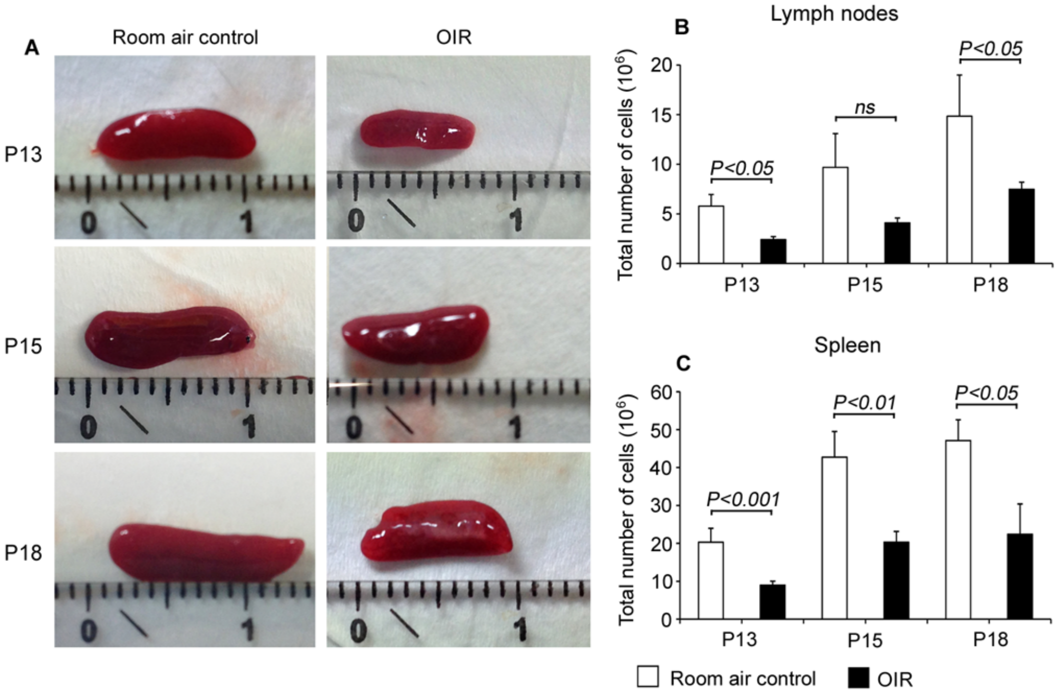

Supplementary Figure 1. Spleens are smaller and have a lower total cell number in OIR.

(**A**). Images of spleens from mice with oxygen-induced retinopathy (OIR) and room air controls at postnatal day (P) 13, P15 and P18. Scale bar is in centimeters. Cell numbers in pooled lymph nodes (**B**) and spleens (**C**) from mice with OIR and room air controls at P13, P15 and P18. *n* = 6 to 27 mice/group. Data are analyzed by a one-way ANOVA with Mann-Whitney U test. ns, not significant. Values are expressed as mean ± s.e.m.

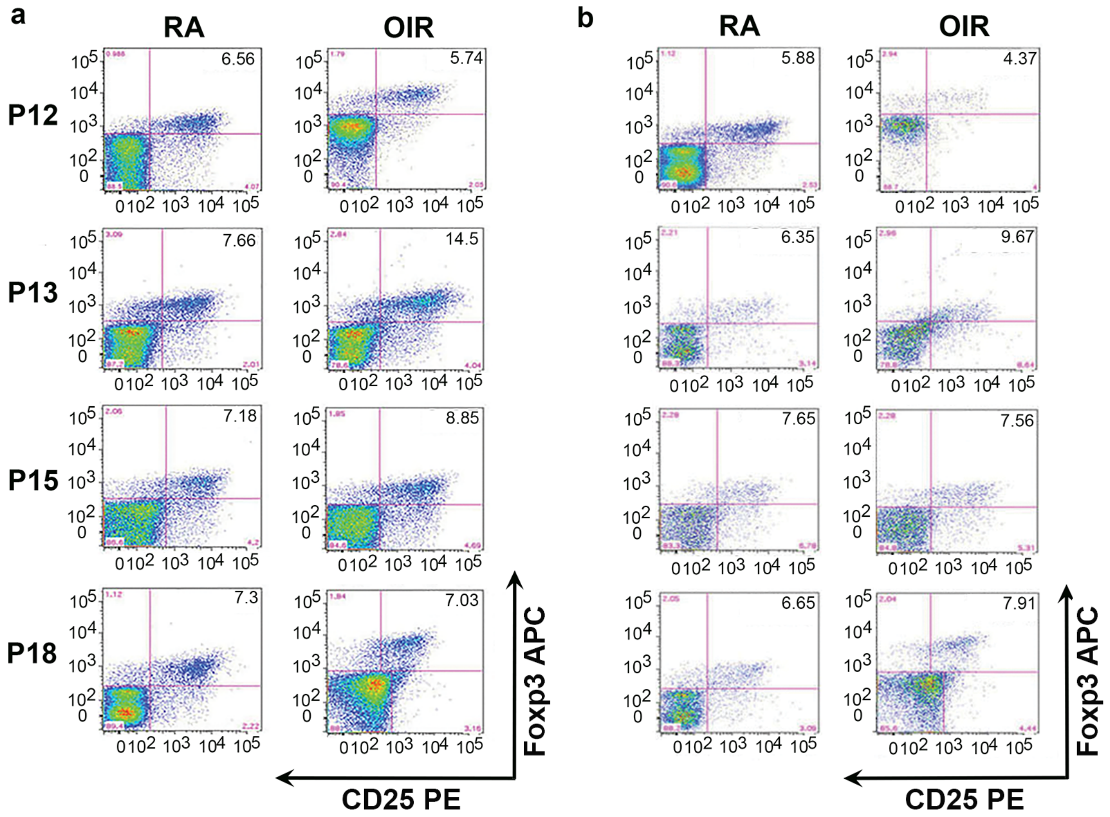

Supplementary Figure 2. Enlarged flow cytometry dot plots from Figure 1. Fluorochromes are phycoerythrin (PE) and allophycocyanin (APC). $CD4^+CD25^+Foxp3^+$ Tregs in pooled lymph nodes (**a**) and spleen (**b**) from C57BL/6J mice with oxygen-induced retinopathy (OIR) and room air controls (RA) at postnatal days (P) 12, 13, 15 and 18.

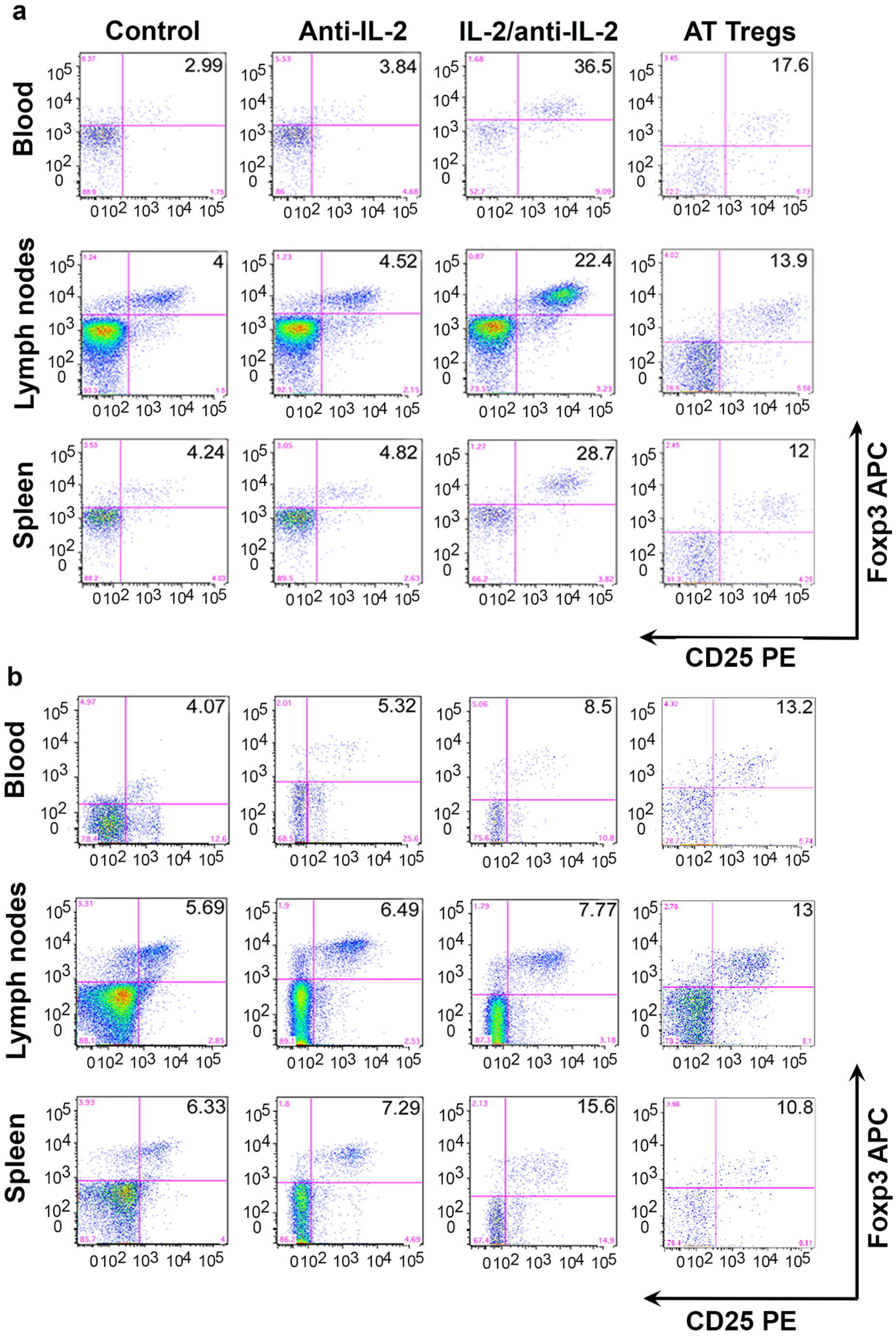

Supplementary Figure 3. Enlarged flow cytometry dot plots from Figure 2. Fluorochromes are phycoerythrin (PE) and allophycocyanin (APC). $CD4^{+}CD25^{+}Foxp3^{+}$ Tregs in blood, pooled lymph nodes and spleen from C57BL/6J mice at postnatal day 12 (**a**) and postnatal day 18 (**b**) with oxygen-induced retinopathy (OIR) and administered an anti-IL-2 control mAb, an IL-2/anti-IL-2 mAb complex and the adoptive transfer (AT) of Tregs.

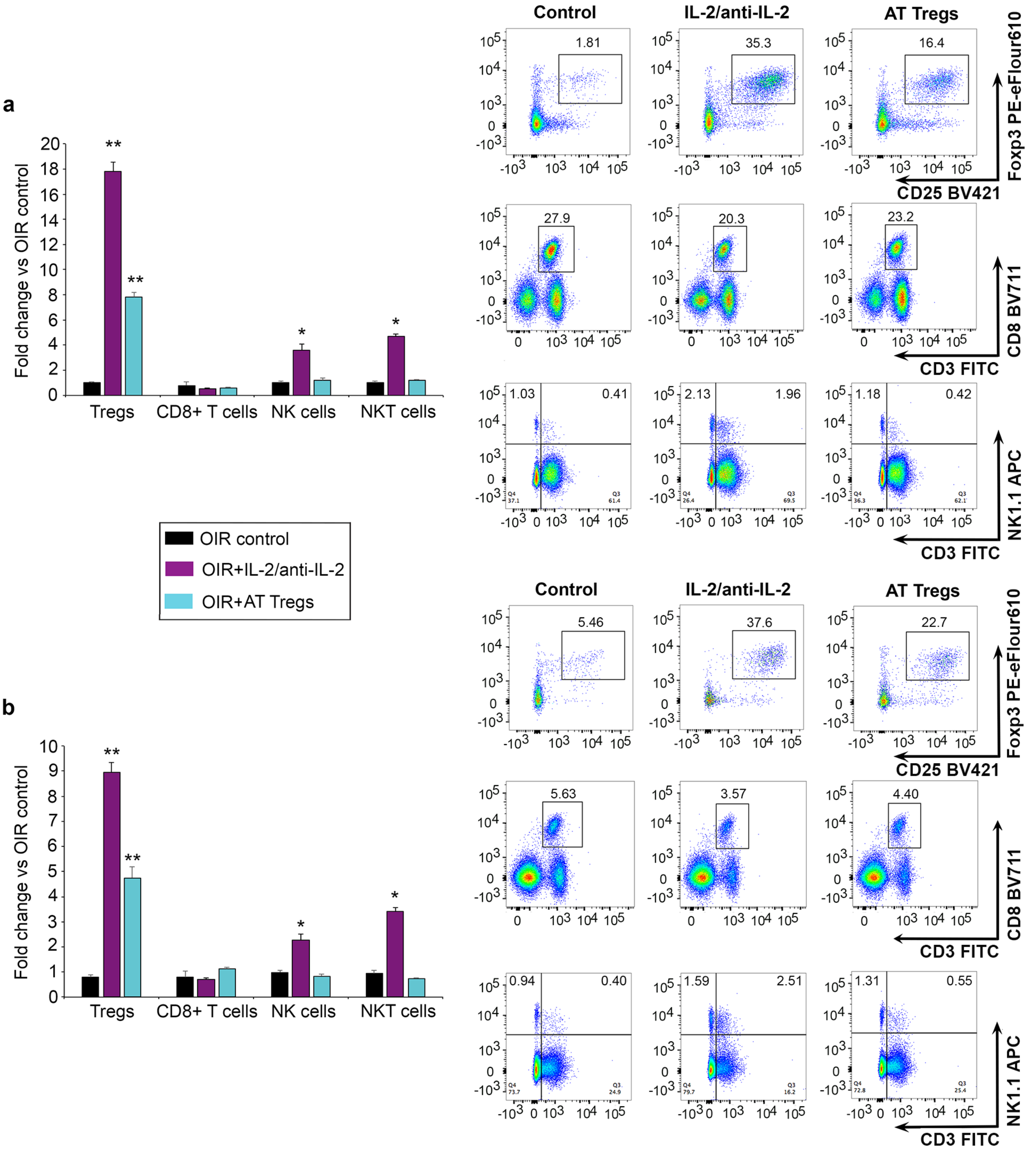

Supplementary Figure 4. Expanding Treg numbers and CD8$^+$ T cells, NK cells and NKT cells. Fluorochromes are phycoerythrin (PE)-eFlour610, allophycocyanin (APC), BV421, BV711 and fluorescein isothiocyanate (FITC). NK, natural killer. NKT, natural killer T cells. Oxygen-induced retinopathy (OIR) mice were treated with the IL-2/anti-IL-2 mAb complex and adoptive transfer (AT) of Tregs. FACS analysis at postnatal day 10 of (**a**) pooled lymph nodes and (**b**) spleen from mice with OIR revealed that the treatments markedly increased Foxp3$^+$ Tregs, had no effect on the number of CD8$^+$ T cells and only the IL-2/anti-IL-2 mAb complex modestly increased NK cells and NKT cells. Data represented as fold change to relevant OIR control (i.e. IL-2 mAb for complex study and effector T cells for adoptive transfer study). *$P < 0.05$ and **$P < 0.01$ to relevant OIR control (one-way ANOVA with Mann-Whitney U test. $n = 5$ mice/group. Values are expressed as mean ± s.e.m.

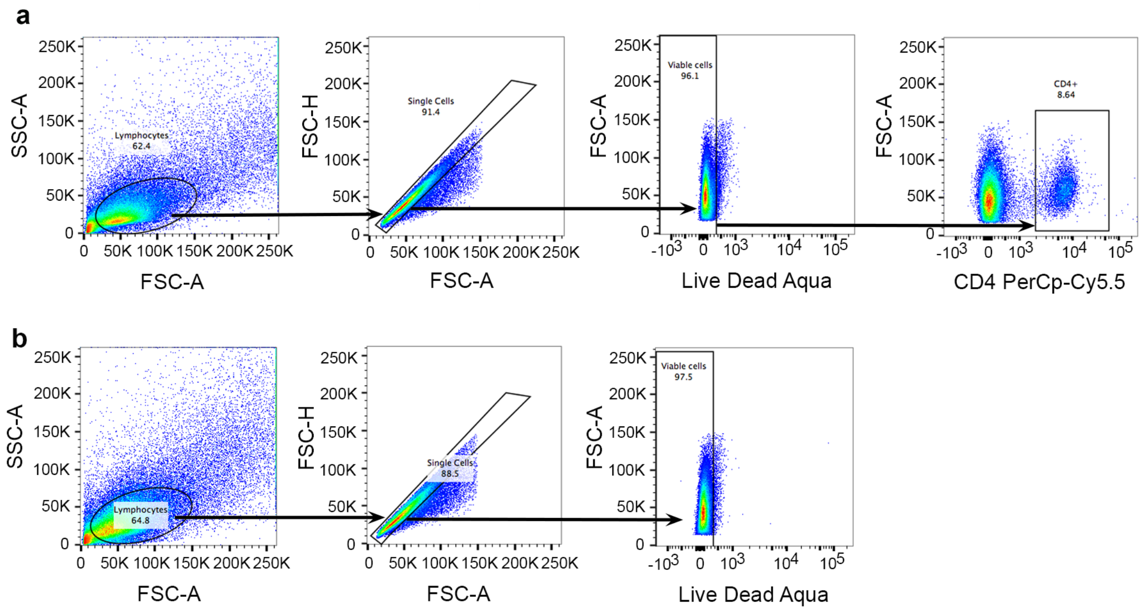

Supplementary Figure 5. Flow cytometry gating strategies. (a) Gating strategy for $CD4^{+}CD25^{+}Foxp3^{+}$ Tregs. **(b)** Gating strategy for $CD8^{+}$ T cells, natural killer cells and natural killer T cells.

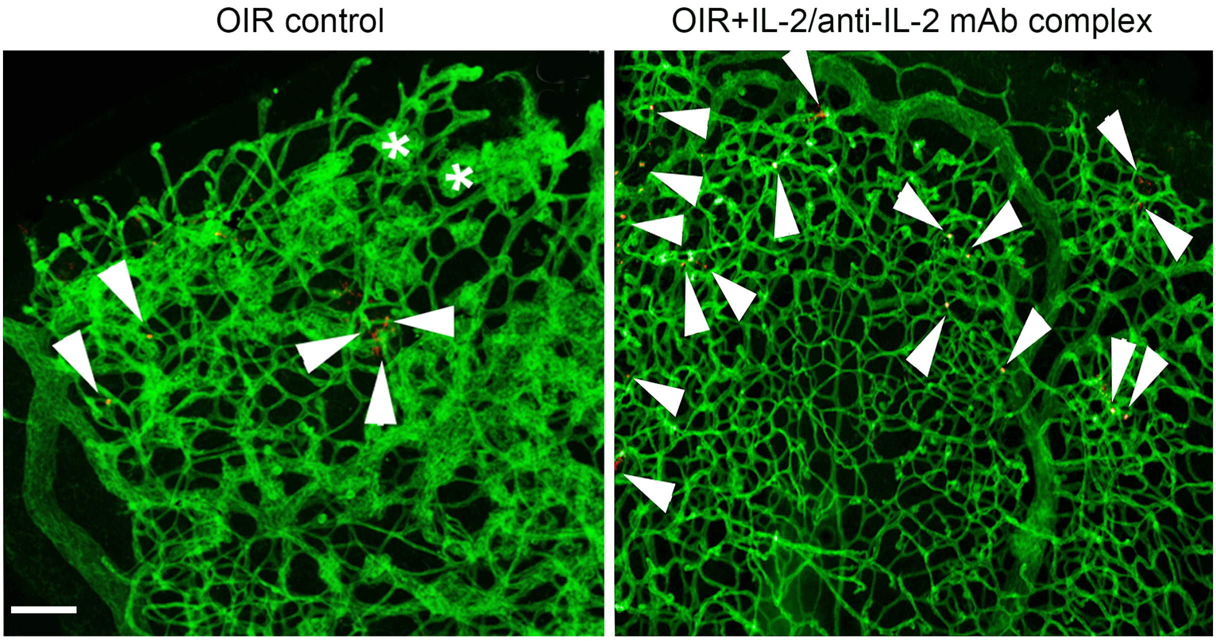

Supplementary Figure 6. Low powered images showing Foxp3^{+} cells in OIR retina. Low powered confocal images from one quadrant of flat mounted retina from Foxp3^{rfp} mice with oxygen-induced retinopathy (OIR) at postnatal day 18. Retina labeled with isolectin (green) to show blood vessels. Foxp3^{+} cells are red (arrowheads). Scale bar, 85 µm. Neovascular tufts (asterisks).

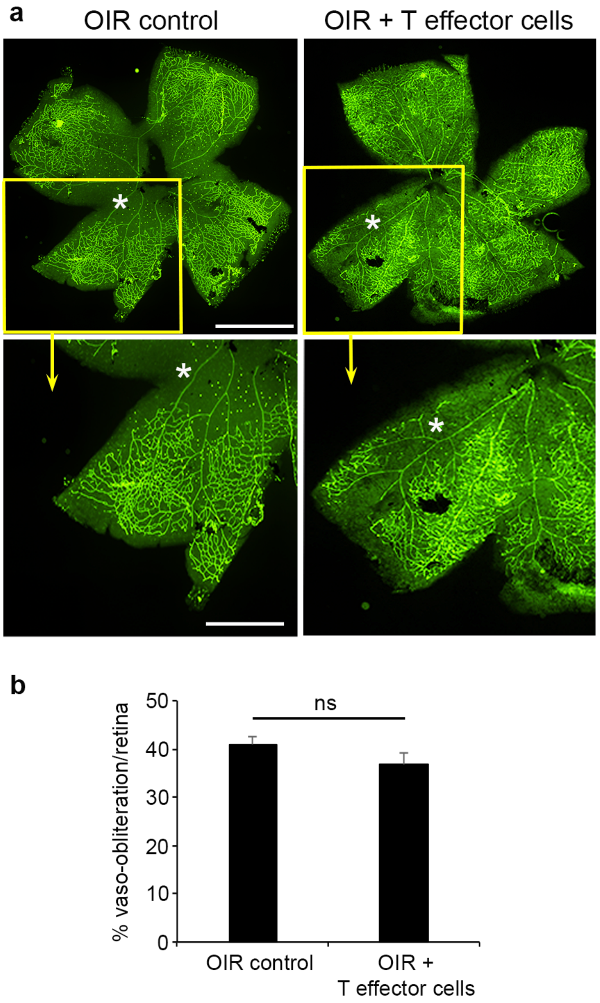

Supplementary Figure 7. Retinal vaso-obliteration following the adoptive transfer of T effector cells. (**a**) Representative confocal images of flat mounted retina labeled with isolectin (green) to show blood vessels and the vaso-obliterated retina (asterisks) in oxygen-induced retinopathy (OIR). Scale bar, 0.5 mm. (**b**) One quadrant (yellow box) is enlarged. Scale bar, 0.25 mm. (**c**) $n = 9$ mice/group with 3 litters/group examined (one-way ANOVA with Mann-Whitney U test). ns, not significant. Values are mean ± s.e.m.

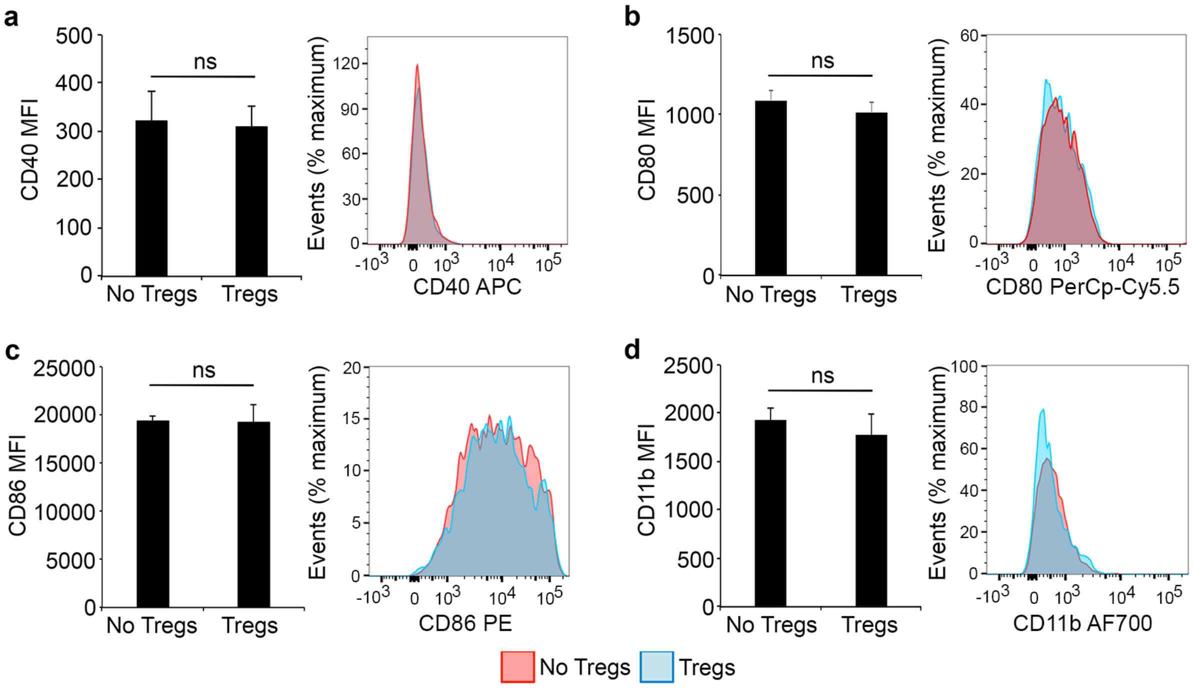

Supplementary Figure 8. Transwell experiments involving Tregs and retinal microglia. Fluorochromes are phycoerythrin (PE), allophycocyanin (APC), AF700 and PerCp-Cy5.5. Tregs were purified from adult C57BL/6J mouse spleen and microglia were obtained from neonatal retina of C57BL/6J mice. Flow cytometry was performed and mean fluorescence intensity (MFI) quantitated for (**a**) CD40, (**b**) CD80, (**c**) CD86 and (**d**) CD11b. ns, not significant. Data are representative of two independent experiments each containing three replicates (one-way ANOVA with a Student's t-test). Values are expressed as mean ± s.e.m.

	P10	*n*	P12	*n*	P13	*n*	P18	*n*
Room air control	NA	NA	6.9±0.1***	28	7.14±0.13***	14	8.4±0.1***	38
OIR control	5.0±0.1	25	5.4±0.1	27	5.59±0.1	15	7.14±0.1	39
OIR + IL-2 mAb	4.7±0.1	26	5.4±0.1	26	NA	NA	7.2±0.1	37
OIR + IL-2/anti-IL-2 mAb complex	5.1±0.1	25	5.3±0.1	26	NA	NA	7.1±0.2	44
OIR+AT Tregs	4.8±0.2	21	5.3±0.1	26	NA	NA	6.9±0.1	41
OIR + AT effector T cells	NA	NA	5.1±0.1	16	NA	NA	NA	NA

Supplementary Table 1. Body weights (g) of mice with OIR and room air controls. P, postnatal day. AT, adoptive transfer. *n*, number of animals. NA, not applicable. Mice with oxygen-induced retinopathy (OIR) have lower body weights than age-matched room air controls and the treatments did not influence body weight. ***$P < 0.001$ to age-matched room air controls (one-way ANOVA with Mann-Whitney U test). Values are mean ± s.e.m.
